# Understanding COVID-19 Vaccine Uptake and Hesitancy in Latinx Sexual and Gender Minority People in North Texas

**DOI:** 10.1007/s12529-025-10395-6

**Published:** 2025-10-03

**Authors:** Sobur Ali, Stacey B. Griner, Malinee Neelamegam, Nathaniel Webb, Nolan Kline

**Affiliations:** 1 Department of Population Health Sciences, College of Medicine, University of Central Florida, Orlando, United States; 2 Department of Population & Community Health, College of Public Health, University of North Texas Health Science Center, Fort Worth, United States

**Keywords:** Vaccine hesitancy, COVID-19, Latinx, Texas

## Abstract

**Background:**

COVID-19 vaccination disparities persist in the US, but few studies focus on Latinx sexual and gender minority (SGM) people. This study aimed to examine the factors shaping COVID-19 vaccine uptake and hesitancy among Latinx SGM people in North Texas.

**Method:**

In a cross-sectional study from June to July 2023, 134 Latinx participants completed a survey on COVID-19 vaccine rate, confidence, and hesitancy. Vaccination rates and hesitancy among SGM and cisgender heterosexual individuals were compared using the chi-square test. Fisher’s exact test was used to examine the association between confidence in vaccine safety and (1) vaccine uptake and (2) vaccine hesitancy.

**Results:**

Among Latinx SGM participants, 27.8% were unvaccinated and 24% were vaccine hesitant. Furthermore, 25.9% demonstrated no likelihood of receiving the vaccine in the future. No significant difference was observed in vaccine uptake and hesitancy between SGM and cisgender heterosexual individuals. Latinx SGM individuals with high confidence in COVID-19 vaccine safety were more likely to be vaccinated than those with low confidence.

**Conclusion:**

Confidence in vaccine safety is strongly correlated with vaccination status, highlighting the need for interventions to build trust and address concerns among Latinx SGM people. Such interventions must focus on intersectional sources of social vulnerability among Latinx SGM people and the diversity of Latinx and SGM identities.

**Supplementary Information:**

The online version contains supplementary material available at 10.1007/s12529-025-10395-6.

## Introduction

Despite safe and effective vaccines, COVID-19 remains a leading cause of death in the United States (US) [1]. Further, disparities in vaccination uptake have remained a significant public health concern, particularly among marginalized populations, including sexual and gender minority (SGM) people [2] and people minoritized based on race and ethnicity, such as Latinx populations [3]. These disparities potentially exacerbate adverse health outcomes for minoritized populations who may experience intersectional vulnerabilities due to their race, ethnicity, and sexual and gender identities.

Latinx and SGM communities face an elevated risk of COVID-19 due to structural inequities, such as limited access to healthcare and higher discrimination rates [4, 5]. For Latinx SGM people, their intersecting racial, ethnic, and gender identities further increase their vulnerability to severe COVID-19 outcomes [6, 7]. Further, existing research has shown that SGM people’s experiences of stigma and medical distrust may influence their COVID-19 vaccine acceptance [8], and that barriers to vaccination include continued medical trauma and perceived emotional violence [9]. Similarly, Latinx individuals may experience elevated rates of vaccine hesitancy due to historic medical distrust and structural vulnerabilities, such as having a precarious immigration status (e.g., undocumented or Deferred Action for Childhood Arrivals recipient) [10]. In the 2023–2024 season, COVID-19 vaccination coverage in the Latinx adult population was among the lowest in the US at 15.3% compared to 24.4% among white non-Hispanic populations and 19.6% Black non-Hispanic populations [11].

Although previous studies have examined vaccination hesitancy among Latinx people [10] and among SGM people [8], there has been limited research on Latinx SGM individuals who may experience compounded intersectional vulnerabilities that inform their COVID-19 vaccine hesitancy. Concerns about vaccination safety and a deep-rooted mistrust in public health initiatives may significantly influence this population’s vaccination decisions [12, 13]. Despite these known challenges, limited research exists on how these compounded obstacles affect vaccine uptake within this population. Our study is therefore informed by growing public health research on intersectionality and the unique ways in which social location and multiple sources of marginalization can converge to shape health-related harms [14].

This study examined the perceptions of vaccine safety and hesitancy among people who identify as SGM and Latinx. First, the vaccine uptake and hesitancy of Latinx SGM individuals compared to cisgender heterosexual (cis-het) individuals was assessed. Second, the reasons for vaccine hesitancy, which focused on safety concerns and side effects, were examined. Moreover, the association between confidence in the COVID-19 vaccine’s safety and the likelihood of vaccination was investigated.

## Methods

### Study population

Data were collected from online surveys of 134 participants from June to July 2023 to examine factors influencing COVID-19 vaccine hesitancy among Latinx people in North Texas who experienced additional social vulnerabilities, such as having a precarious immigration status, identifying as SGM, and having recently experienced pregnancy. North Texas comprises the Dallas-Fort Worth (DFW) Metroplex (the fourth most populous metropolitan area in the United States) and surrounding areas with a population of over 8.5 million people. The area is home to a diverse population; in the DFW area, 42% of residents identify as non-Hispanic white, 29% as Hispanic or Latino, 16% as Black or African American, and 7% as Asian American [15].

Participants were recruited through (1) training a local Latinx SGM community member who engaged in snowball sampling from their social network and (2) online via a survey panel provider. Surveys were administered through a Qualtrics link and were available in English or Spanish based on participant preference. To be included in the study, respondents had to speak English or Spanish, be age 18 or older, identify as Latinx, and identify as having one of the three statuses of social vulnerability of focus: having a precarious immigration status, identifying as SGM, or having recently experienced pregnancy. Pre-screening questions ensured participants were eligible, and consent was obtained before survey participation.

### Measures

The measures were derived from a Common Survey questionnaire created by the National Institutes of Health Community Engagement Alliance against COVID-19 (CEAL). CEAL initiatives were nationwide, and varying projects and standardized metrics were established to facilitate comparable assessment procedures among participating states. The outcomes of this study were self-reported COVID-19 vaccine uptake and vaccine hesitancy among Latinx SGM people, and the predictor variable was confidence in the safety of the COVID-19 vaccine. Participants were asked to self-report their gender and sexual identity. Inclusion criteria were (1) being aged 18 or older, (2) able to speak English or Spanish, (3) residing in North Texas, and (4) identifying as Latinx.

#### COVID-19 Vaccine Uptake

Participants responded to a standardized vaccine uptake question: “Have you received at least one dose of the COVID-19 vaccine?” Response options included: “No, have not gotten the vaccine,” “Yes, received the first dose of two-dose vaccine,” “Yes, received one dose of the vaccine,” “Yes, received both doses of the two-dose vaccine,” “Yes, got both doses of two-dose vaccine and a booster,” and “Yes, received one dose of the vaccine and a booster.” Vaccine uptake was dichotomized into two groups: unvaccinated (No, have not gotten the vaccine) and vaccinated (at least received one dose of the vaccine). Unvaccinated individuals were next asked why they had not received a COVID-19 vaccine.

#### COVID-19 Vaccine Hesitancy

Participants were asked to respond to a 10-item scale about their confidence in COVID-19 to measure vaccine hesitancy [16]. A 5-point Likert scale was used to map responses for each (1 = strongly agree, 2 = agree, 3 = neither agree nor disagree, 4 = disagree, 5 = strongly disagree), such that a higher value indicates greater hesitancy. Of the ten questions, three negative-worded questions were reverse-coded. The vaccine hesitancy score (VHS) was calculated as an average and defined “COVID-19 vaccine hesitancy” as a VHS score > 3, following previous studies [17, 18].

#### Covariates

Covariates included respondent demographics, including age, language, race/ethnicity, and gender, which were collected with predefined questions and responses (Supplementary Table 1). The respondents were asked about their confidence that the COVID-19 vaccines currently available in the US are safe. Response options included “not at all confident,” “not too confident,” “somewhat confident,” and “very confident.” These responses were categorized into two groups: low confidence (not at all confident and not too confident) and high confidence (somewhat confident and very confident).

### Statistical Analysis

A chi-square test was conducted to examine the difference in vaccine intake and vaccine hesitancy in SGM groups compared to cis-het individuals. Fisher’s exact test was conducted to examine the relationship between the confidence level of vaccine safety, vaccine intake, and vaccine hesitancy. A result returning a *p*-value < 0.05 was considered statistically significant. All analyses and visualizations were conducted using R statistical software.

## Results

The median age of the participants was 31 years (range, 18–66 years), and the interquartile range (IQR) was 15. Participants identifying as bisexual, gay, lesbian, and others were categorized as a sexual minority (SM, *n* = 54), while those identifying as heterosexual and straight were combined as cis-het (*n* = 80). Five individuals who identified as sexual minorities also identified as gender minorities (GM; four nonbinaries, one transgender), and for this reason, the overall sample was categorized as a SGM group. The median age of the SGM group was 29 years (IQR 11.8), and the cis-het group was 32 years (IQR 16.5).

Among the SGM participants, 28.8% were not vaccinated, whereas 20% were not vaccinated among the cis-het group. In terms of vaccine hesitancy, 24% of the SGM group were vaccine hesitant, while 20% of the cis-het group were vaccine hesitant. No significant difference between the two groups was observed in demographic characteristics, vaccination status, and vaccine hesitancy (Table 1).

**Table 1:** Summary characteristics of participants by sexual and gender orientation

Variables	Sexual and gender minorities^a^	Cisgender heterosexual^b^	*p*-value
Participants *n* (%)	54 (40.3%)	80 (59.7%)	
Median age (IQR^c^)	29 (11.8)	32 (16.5%)	0.18
Education			0.60
Below high school	5 (9.4%)	9 (11.3%)	
College and above	10 (18.8%)	20 (25.3%)	
High school	38 (71.7%)	50 (63.2%)	
Income			0.87
Less than $35k	21 (38.8%)	35 (44.3%)	
$35k–$75k	17 (31.4%)	24 (30.3%)	
Above $75k	12 (22.2%)	16 (20.2%)	
Prefer not to answer	4 (7.4%)	4 (5.1%)	
Vaccinated			0.40
Yes	39 (72.2%)	64 (80.0%)	
No	15 (28.8%)	16 (20.0%)	
Vaccine hesitancy			0.73
Yes	13 (24.1%)	16 (20.0%)	
No	41 (75.9%)	64 (80.0%)	

^a^Bisexual, *n* = 29; gay, *n* = 6; lesbian, *n* = 12; other sexual orientation, *n* = 7

^b^Straight, *n* = 66; cisgender heterosexual, *n* = 14

^c^Interquartile range

**Fig. 1:**
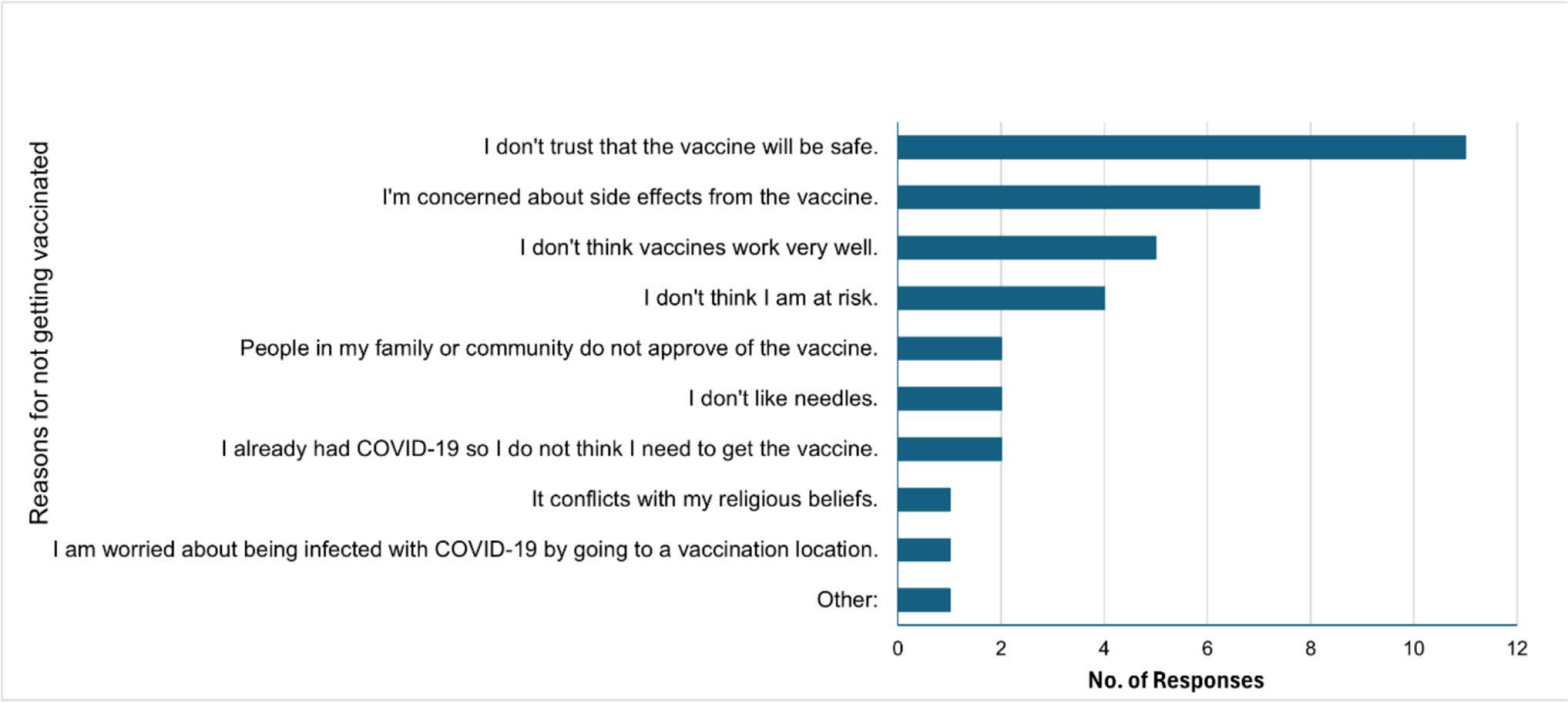
Reasons for not getting vaccinated among Latinx sexual and gender minority participants (*n* = 15). The bar graph illustrates the frequency of reported reasons for vaccine refusal

Unvaccinated SGM individuals were further asked how likely they would get a COVID-19 vaccine in the next 3 months. The majority (93%) were unlikely to get vaccinated in the next 3 months. Among 15 unvaccinated SGM participants, 11 responded that they do not trust that the vaccine will be safe, seven responded that they are concerned about the side effects of the vaccine, and five responded that they don’t think vaccines work very well (Fig. 1).

**Fig. 2:**
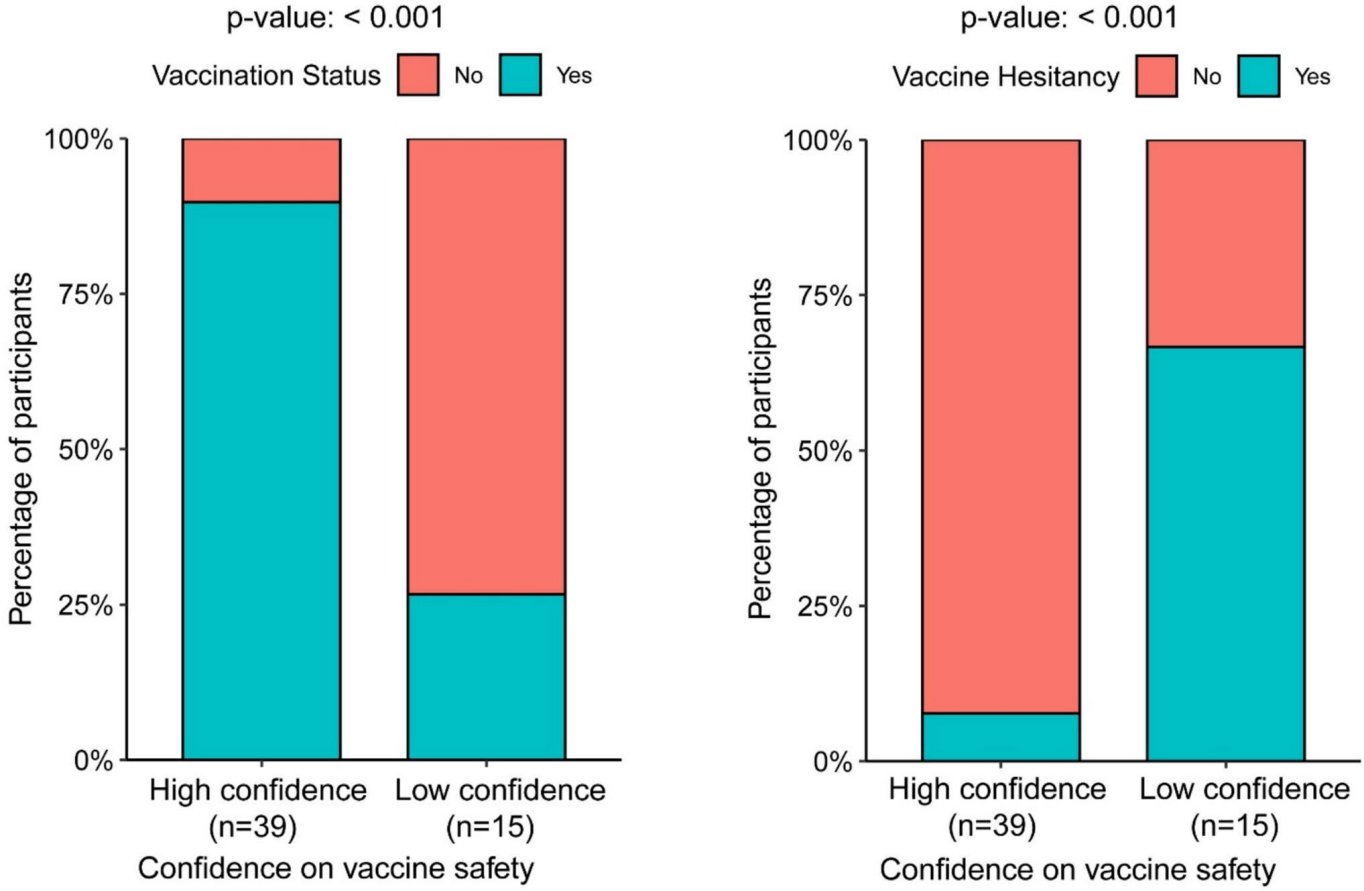
Comparison of SGM participants’ COVID-19 vaccine intake and hesitancy based on confidence in COVID-19 vaccine safety. Fisher’s exact test was used to compare the study population with high confidence in vaccine safety with those with low confidence in vaccine safety regarding vaccine intake and hesitancy. The data represent categorical response frequencies from the study population only

Further, the association between participants’ confidence in COVID-19 vaccine safety and their vaccine uptake and hesitancy status was investigated. Among Latinx SGM participants, 71.2% showed high confidence, and 28.8% showed low confidence in COVID-19 vaccine safety. Most participants with high confidence in COVID-19 vaccine safety were vaccinated and non-hesitant. Among 15 participants with low confidence in COVID-19 vaccine safety, 11 did not receive the COVID-19 vaccine, and 10 showed vaccine hesitancy. It was observed that individuals with high confidence that the COVID-19 vaccine is safe are more likely (OR, 21.8; 95% CI 4.2–150.1; *p*-value, < 0.001) to have received the COVID-19 vaccine than those with low confidence. Individuals with high confidence in the COVID-19 vaccine are approximately 97% less likely to be vaccine hesitant than those with low confidence, with an odds ratio of 0.032 (95% CI, 0.003–0.206; *p*-value, < 0.001) (Fig. 2).

## Discussion

This study highlights findings from a COVID-19 vaccine intake and vaccine hesitancy within Latinx SGM communities. The lack of differences in vaccination rates for an understudied community (SGM Latinx people) may indicate a possible “floor” in vaccine hesitancy for some minoritized groups. Since this study examined SGM Latinx people, it is possible that vaccine hesitancy influenced by one minoritized life experience (e.g., being Latinx) was not necessarily compounded by another minoritized life experience (e.g., having an SGM identity). In other words, rather than seeing compounded impacts of intersectional life experiences, we did not see any significant differences in vaccine hesitancy for SGM people compared to the broader sample of Latinx individuals. Additional research may be needed to determine if a vaccine hesitancy floor effect may be present for SGM populations who are also minoritized due to their ethnicity. Although there was no significant difference in vaccination status or hesitation between sexual minority and cis-het populations, the research indicates major distrust in vaccine safety and efficacy among the unvaccinated SGM people. Additional research examining why such distrust exists among this group is needed.

A large proportion of unvaccinated Latinx SGM participants indicated hesitance to receive vaccinations in the next 3 months, highlighting a need for interventions to address concerns that inform vaccine hesitancy. The primary reasons identified for vaccination reluctance—distrust in vaccine safety, concerns regarding side effects, and skepticism about vaccine efficacy—align with prior studies on vaccine hesitancy in marginalized populations [19, 20]. The results indicate broader social issues while suggesting that some Latinx SGM people may hesitate receiving the COVID-19 vaccine, possibly due to experiences of marginalization and skepticism towards healthcare systems. Resolving such mistrust requires an integrated approach that incorporates trust-building, sharing accurate information, and the assurance of equitable healthcare access [21, 22].

Addressing mistrust among Latinx SGM people may need to consider the intersectional vulnerabilities of this population. For example, efforts to improve vaccine trust among people who are Latinx and SGM and have a precarious immigration status must consider the unique positions of undocumented immigrants or DACA recipients, whose immigration statuses and SGM identities may add layered vulnerabilities informing vaccine hesitancy. Trust in vaccine-related information can, therefore, be complicated by multiple intersecting social vulnerabilities shaping some Latinx SGM people’s social positions.

A noteworthy finding of this study is the association between confidence in COVID-19 vaccine safety and vaccine uptake. Individuals exhibiting high confidence in the safety of COVID-19 vaccinations demonstrated a significantly increased likelihood of vaccination, while those possessing greater confidence were less prone to vaccine hesitancy. This highlights the importance of confidence in vaccination safety in promoting vaccine uptake and reducing hesitation.

Moreover, the study’s results emphasize the necessity of tailoring public health communications for specific demographics. While our sample did not show meaningful differences in vaccine hesitancy or vaccination status, the results nevertheless serve as a reminder of the importance of public health messages that resonate with populations experiencing varying forms of social marginalization. Existing scholarship underscores how SGM populations face heightened individual-level, social, and structural challenges that uniquely shape their COVID-19 vulnerabilities [23, 24]. Future studies may consider if targeted messaging that specifically addresses the interests and concerns of SGM communities can enhance immunization rates and mitigate health inequalities. The research also emphasizes the necessity for continuous monitoring and assessment of vaccination initiatives to recognize and address emerging issues. As the pandemic progresses, it is crucial to modify tactics to guarantee that all individuals, inclusive of their sexual orientation or gender identity, possess fair access to COVID-19 vaccines and the necessary knowledge to make informed health decisions.

### Limitations

This study has some limitations that warrant acknowledgment. The sample size was relatively small, especially for the SGM group, limiting the findings’ generalizability to the broader Latinx SGM community. Subsequent research should use more extensive and more heterogeneous samples to validate these results. Furthermore, our research depended on self-reported vaccination status and vaccine hesitancy, which may be influenced by recall bias and underreporting. Additionally, the cross-sectional nature of this study precludes causality. Lastly, our study focused on people in North Texas, the fourth largest metropolitan area in the US, but it represents one region of the US, which limits potential generalizability. Despite these limitations, this study offers meaningful insights into the barriers to vaccination encountered by Latinx SGM people, an underrepresented demographic in public health research.

## Conclusion

Our research underscores ongoing vaccine hesitancy within some Latinx SGM people, driven mainly by vaccine safety and efficacy concerns. Vaccination rates among SGM people and cis-het individuals were comparable, and additional research about this comparability may be needed to examine the possibility of a vaccine hesitancy floor among minoritized people, or the potential effects of resilience among people with multiple intersecting vulnerabilities. Existing research has described the unique vulnerabilities of SGM people that may structure COVID-19 vaccine hesitancy and vaccination disparities [24]. Future research on possible differences informing vaccine hesitancy may be needed, especially to increase vaccine trust. Enhancing vaccine trust is essential for improving uptake, especially among populations that experience multiple forms of social marginalization. While the World Health Organization officially ended the COVID-19 public health emergency of international concern, continued vaccine hesitancy suggests that needed efforts to work towards equitable health outcomes can be made for all.

## Supplementary Information

Below is the link to the electronic supplementary material.

Supplementary Material 1 (DOCX  17.7 KB)

## Data Availability

Data will be made available on request.
