# Supplementary material for: Understanding COVID-19 Vaccine Uptake and Hesitancy in Latinx Sexual and Gender Minority People in North Texas
Source: Int J Behav Med. 2025 Oct 3;33(2):316–22. doi: 10.1007/s12529-025-10395-6 (PMC13161339; doi:10.1007/s12529-025-10395-6)
Supplement: Supplementary file 1 — Supplementary Material 1 (DOCX 17.7 KB) [file 12529_2025_10395_MOESM1_ESM.docx]

**Supplementary Table 1:** **Questions and response options for measures of gender identity, sexual identity, and race and ethnicity**

**What is your gender?**

- Man
- Woman
- Transgender Female or Trans Woman
- Transgender Male or Trans Male
- Nonbinary, Genderqueer, or Genderfluid
- I would describe my gender as: _______________
- Prefer not to answer

**Which of the following best describes how you think of yourself?**

- Gay
- Lesbian
- Straight (that is, not gay, lesbian, or bisexual)
- Bisexual
- Other
- Prefer not to answer

**Are you of Hispanic or Latino origin?**

- No
- Yes
- Prefer not to answer

If yes, which of the following best describes your Hispanic/Latino heritage?

- Cuban
- Colombian
- Dominican
- Guatemalan
- Honduran
- Mexican or Mexican American or Chicano
- Puerto Rican
- Salvadoran
- Specify: (for example, Ecuadorian, Nicaraguan, Peruvian, Spaniard, Venezuelan) ______________
